# Supplementary material for: Condensation-dependent interactome of a chromatin remodeler underlies tumor suppressor activities
Source: Nat Commun. 2025 Oct 30;16:9599. doi: 10.1038/s41467-025-64655-w (PMC12575849; doi:10.1038/s41467-025-64655-w)
Supplement: Supplementary file 1 — Supplementary Information [file 41467_2025_64655_MOESM1_ESM.pdf]

Supplementary Figures

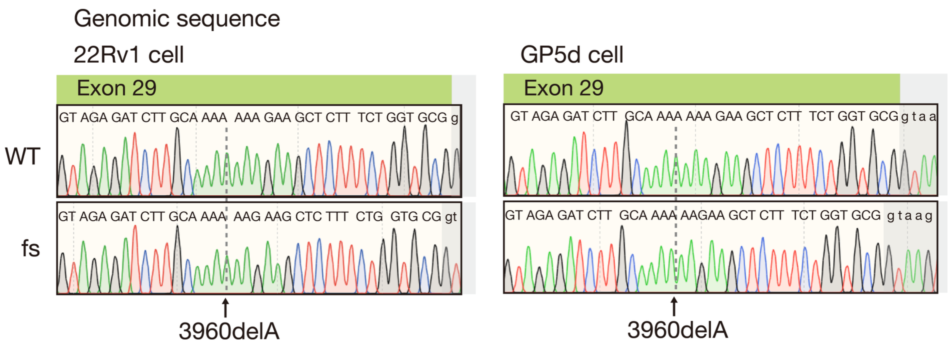

Supplementary Fig. 1 | Genomic sequence of CHD1 showing monoallelic E1321fs mutation in 22Rv1 prostate (left) and GP5d colon (right) cancer cells.

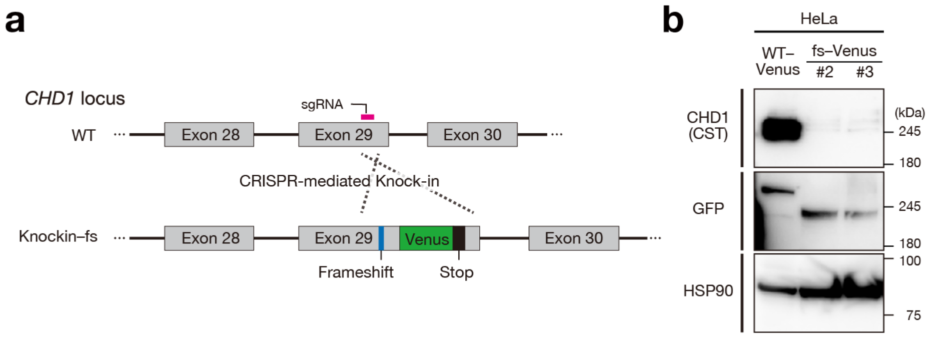

Supplementary Fig. 2 | Expression of the CHD1^{E1321fs}-Venus knock-in protein suggests that it might be resistant to mRNA decay mechanisms. a, The schematic outlines the strategy used for generating HeLa cells with knock-in *CHD1^{E1321fs}-Venus*. **b**, Western blotting using anti-CHD1 antibody (which specifically detects CHD1^{WT}) and anti-Venus antibody.

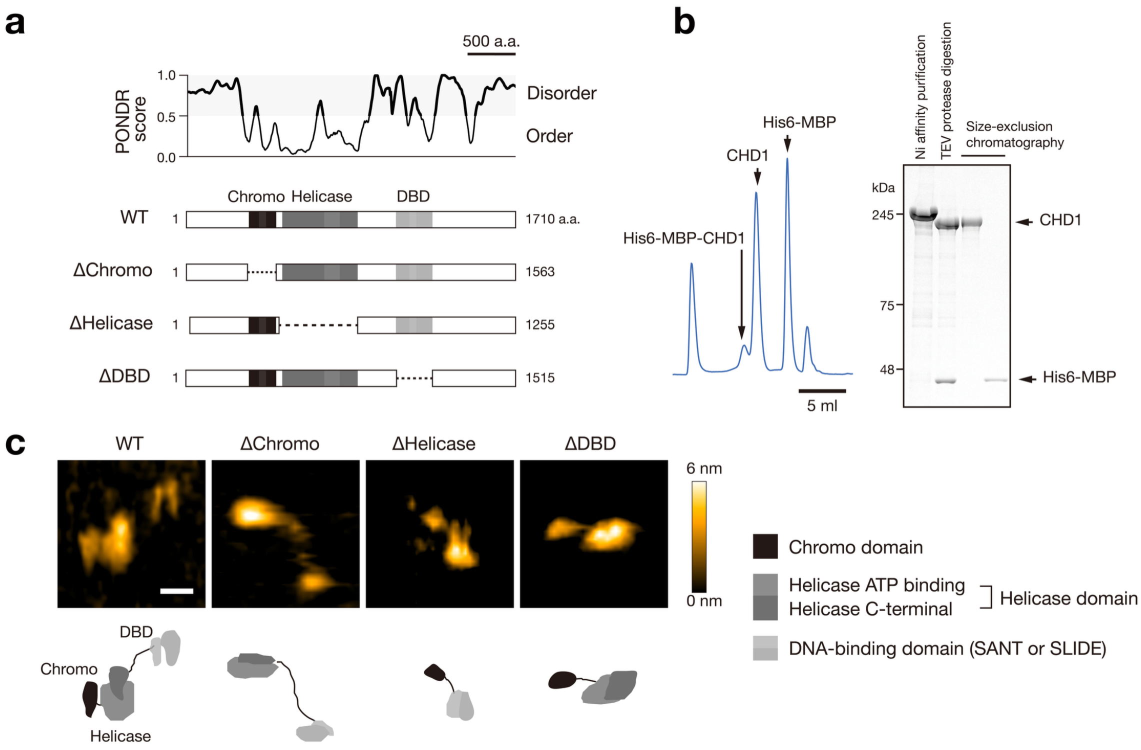

Supplementary Fig. 3 | Domain-deleted CHD1 variants visualized by HS-AFM. a, PONDR score analysis of CHD1 (upper) and schematic representation of CHD1^{WT} and domain-deleted variants. **b**, Elution profile of CHD1 on size exclusion chromatography after TEV digestion (left), followed by analysis using SDS-PAGE and Coomassie Brilliant Blue staining (right). **c**, HS-AFM results showing domain assignment in CHD1 variants through single-molecule live imaging. Scale bar, 10 nm. Schematic diagrams depicting the domains of domain-deleted variants.

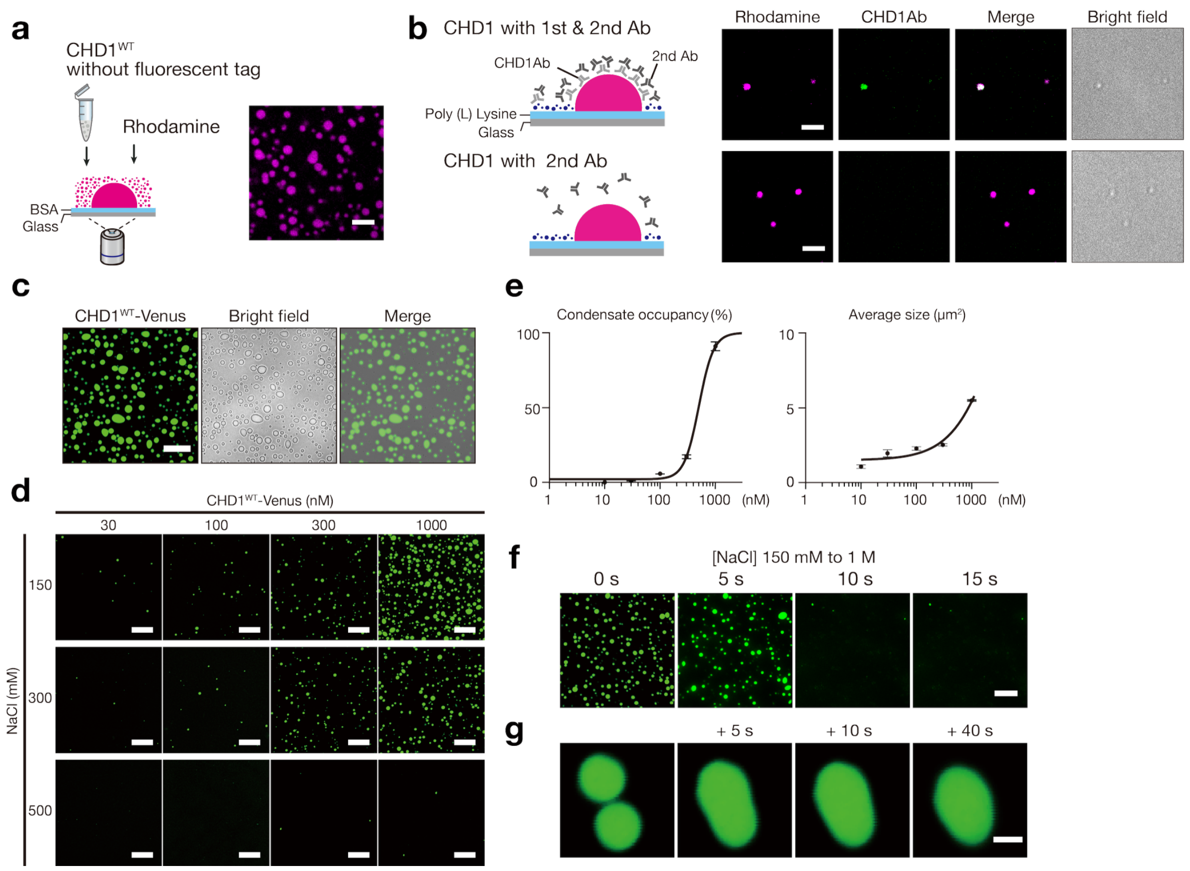

Supplementary Fig. 4 | *In vitro* condensation properties of CHD1^{WT}. a, Droplet assay of CHD1^{WT} without fluorescent tag, visualized using Rhodamine B staining, to demonstrate the condensation property in the absence of a fluorescent tag. Scale bar, 20 µm. **b**, Immunostaining-based droplet assay of CHD1^{WT} without a fluorescent tag, further confirming its condensation behavior. Scale bars, 5 µm. **c**, Droplet assay of CHD1^{WT}–Venus, utilizing bright-field images for size determination. Scale bar, 20 µm. **d**, Droplet assay results showing concentration-dependent and salt-dependent formation of CHD1 condensates. Scale bars, 20 µm. **e,** The occupancy (left) and average size (right) of CHD1^{WT}-Venus condensates in Droplet A buffer containing 150 mM NaCl were quantified within a 4.51 × 10^{-2} mm^2 area. Three fields from independent sessions were randomly selected for analysis. **f, g,** Live imaging of CHD1–Venus condensates exhibiting deformation upon exposure to 1M NaCl (scale bar, 20 µm) (**f**) and transformation and fusion events (scale bar, 2 µm) (**g**). Source data are provided as a Source Data file.

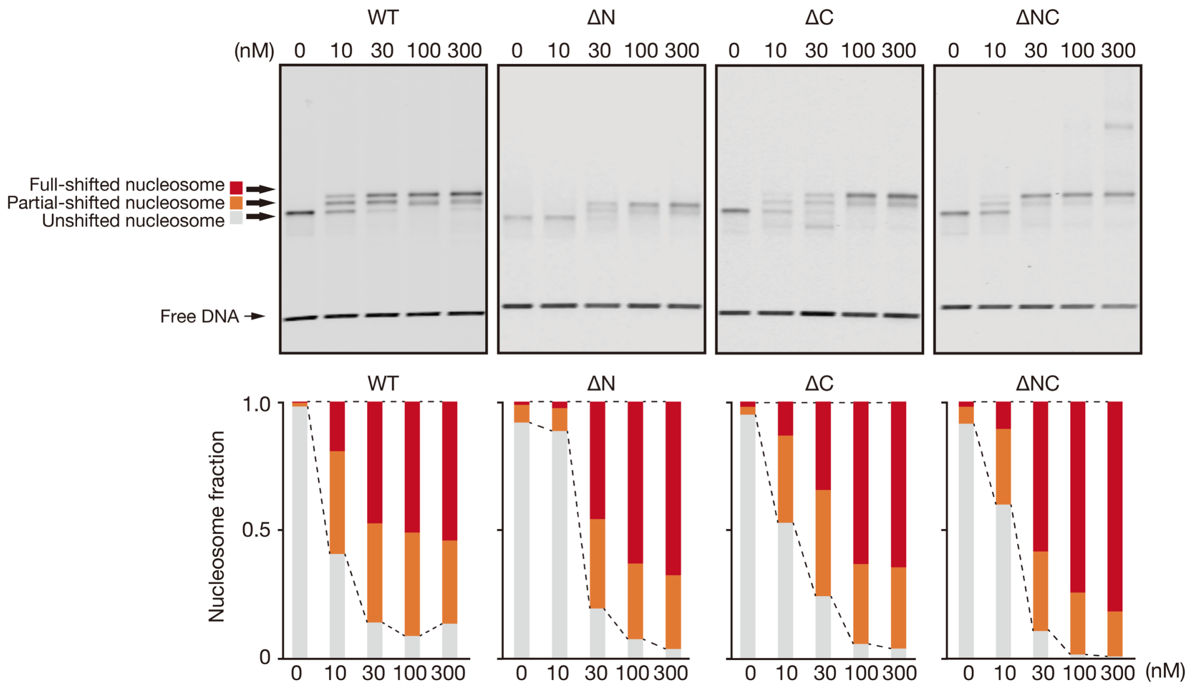

Supplementary Fig. 5 | IDR of CHD1 is not required for nucleosome sliding. The nucleosome sliding assay was performed using a fixed concentration of nucleosomes (10 nM) and varying concentrations of CHD1 proteins as indicated. Representative images (top) and averages of shifted nucleosome fractions (bottom, $n = 2$). Source data are provided as a Source Data file.

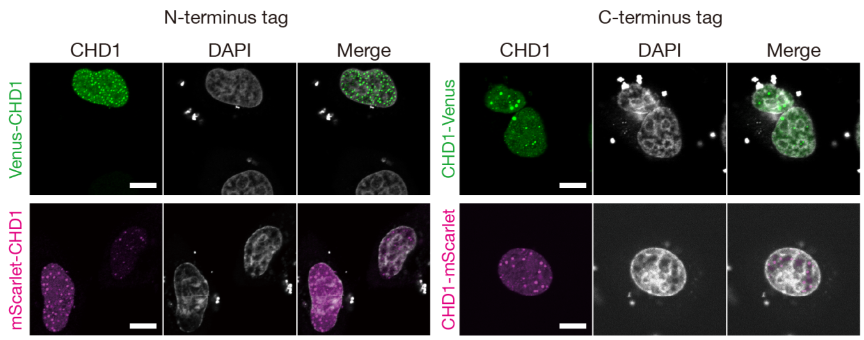

Supplementary Fig. 6 | Tag does not affect the condensation behavior of CHD1^{WT}–Venus in cells. Fluorescence images of HeLa cells expressing CHD1^{WT} tagged with Venus or mScarlet at either the N- or C-terminus. Scale bars, 10 µm.

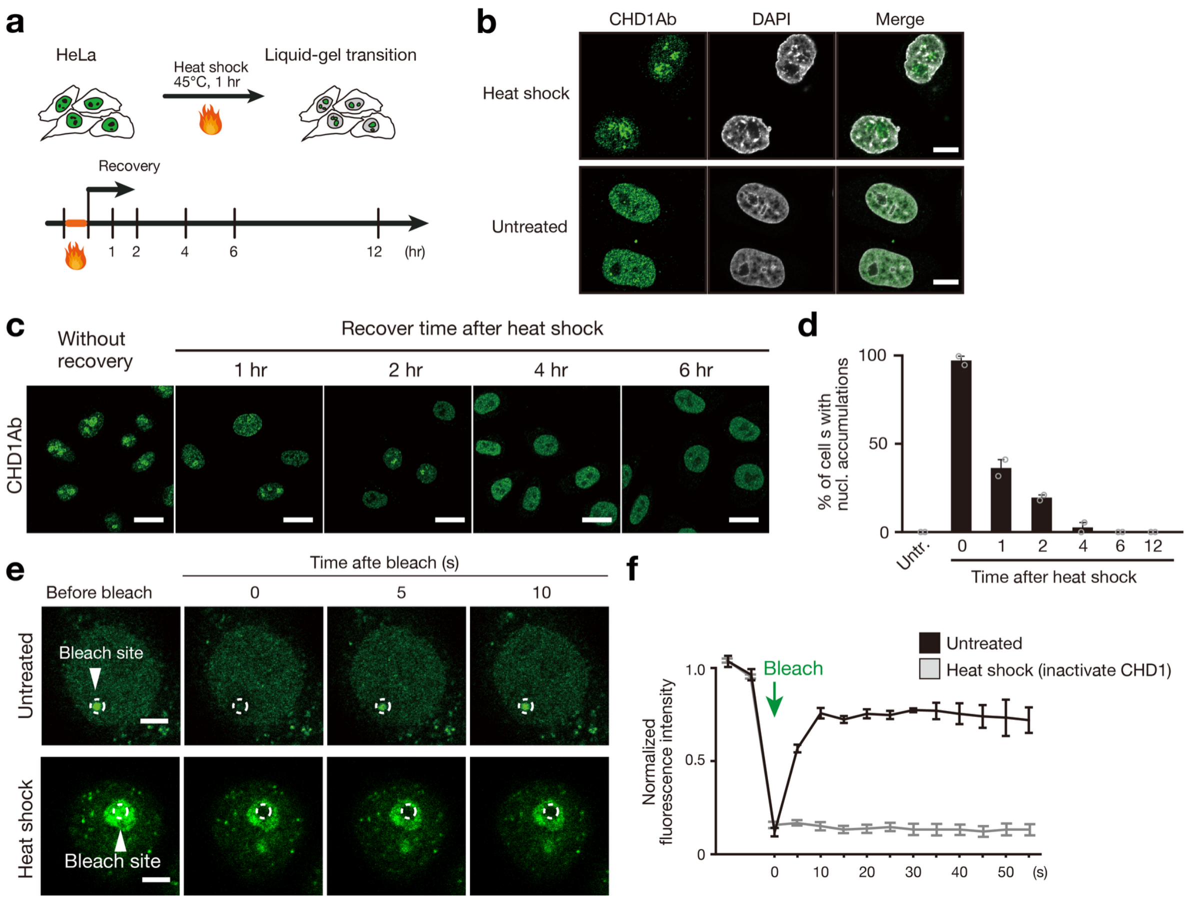

Supplementary Fig. 7 | Heat shock induces CHD1 to form more stable gel-like condensates, distinct from its dynamic native state. a, Experimental design of heat shock experiment. **b**, Heat shock induces large amorphous CHD1 condensates in nucleoli. Confocal images of untreated or heat-shocked (1 h, 45°C) cells stained with an anti-CHD1 antibody. Scale bars, 10 µm. **c**, Confocal images of CHD1-Venus KI HeLa cells fixed at indicated time points after heat-shock recovery. Scale bars, 20 µm. **d**, Percentage of HeLa cells with CHD1 nucleolar accumulation. Data are presented as the mean ± SEM, *n* = 2 independent experiments. **e**, Confocal images of Spot-FRAP analysis of CHD1-Venus in asynchronous cells (upper) or heat-shocked cells (lower). Scale bars, 5 µm. **f**, FRAP signal in the bleached area before and after bleaching. The bleaching intensity was the same in each experiment. Data are presented as the mean ± SEM, *n* = 3 independent experiments. Source data are provided as a Source Data file.

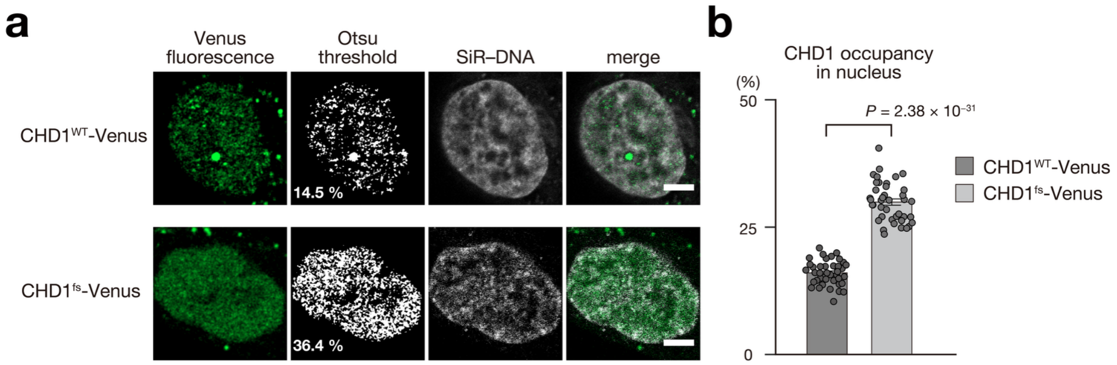

Supplementary Fig. 8 | Nuclear condensation of CHD1^{WT}−Venus and CHD1^{E1321fs}−Venus. a, Live cell imaging of HeLa cells with knock-in of either *CHD1^{E1321fs}−Venus* or *CHD1^{WT}−Venus*. Nuclei were stained with Silicon Rhodamine (SiR)−DNA for live-cell imaging. The Otsu thresholding method was utilized to derive binary values from fluorescence intensity, enabling quantification of CHD1 occupancy in the nucleus. Scale bars, 5 µm. **b**, Forty cells were analyzed from two clones. Each cell's percentage was plotted as a dot and analyzed using an unpaired Student's t-test. Source data are provided as a Source Data file.

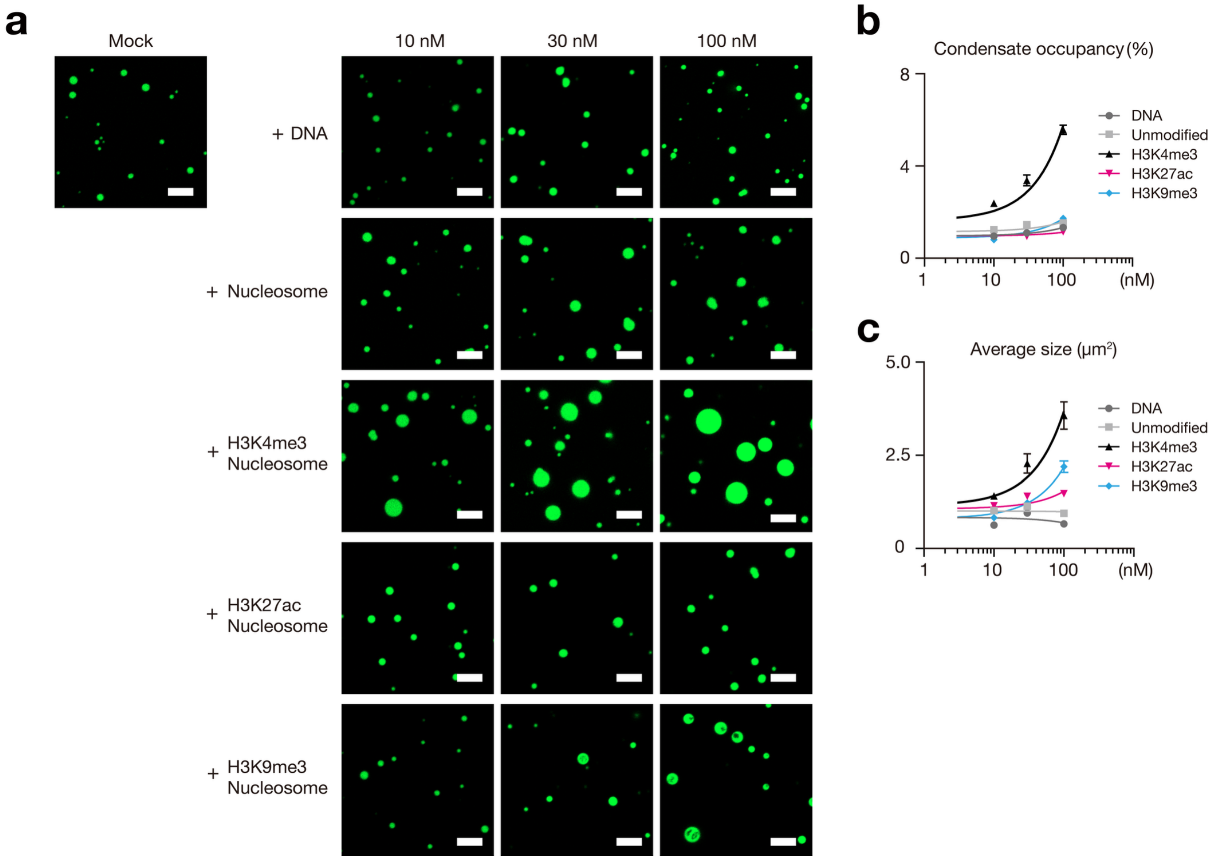

Supplementary Fig. 9 | H3K4me3-modified nucleosomes promote CHD1 condensation in a dose-dependent manner. **a**, Confocal images of CHD1^{WT}-Venus condensates. 30 nM of CHD1^{WT}-Venus was mixed with DNA or nucleosomes at the indicated concentration. Scale bars, 5 μm. **b**, **c**, The occupancy (**b**) and average size (**c**) of CHD1^{WT}-Venus condensates in 1.03 × 10^{-2} mm^2 area. Seven fields were randomly selected. Source data are provided as a Source Data file.

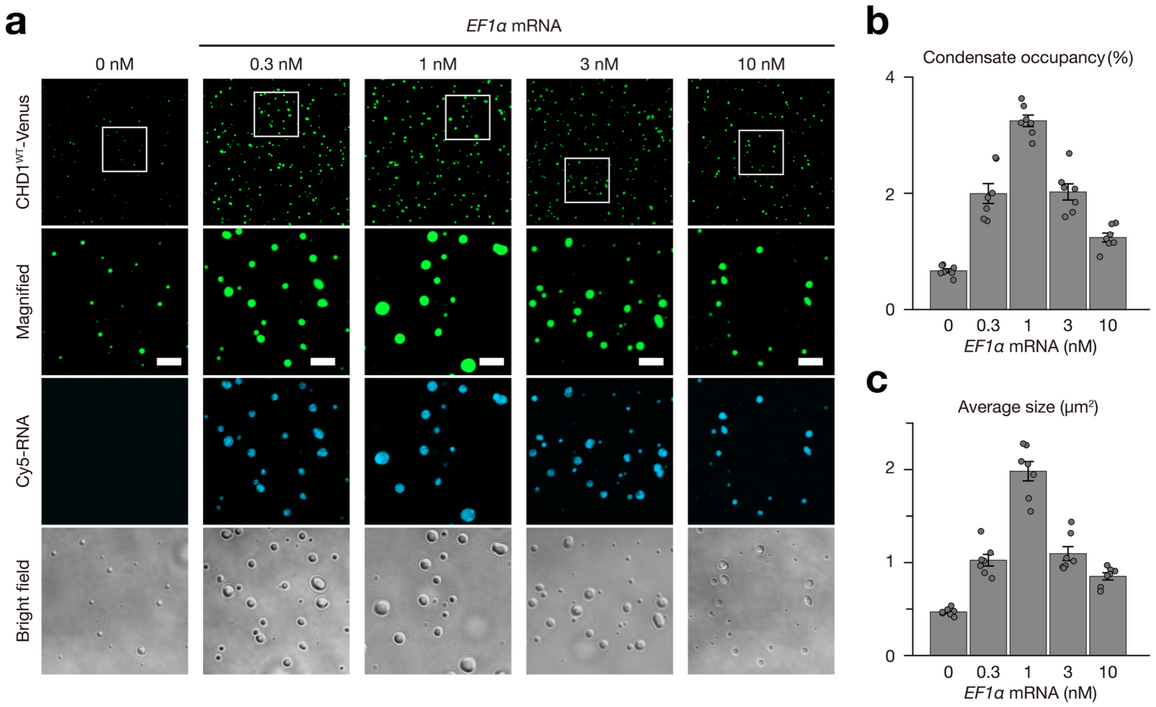

Supplementary Fig. 10 | RNA promotes CHD1 condensation in a dose-dependent manner. a, Confocal images of droplet assay at indicated concentration of RNA. Scale bars, 5 μm. **b**, **c**, The occupancy (**b**) and average size (**c**) of $CHD1^{WT}$-Venus condensates in 1.03×10^{-2} mm^2 area. Seven fields were randomly selected. Source data are provided as a Source Data file.

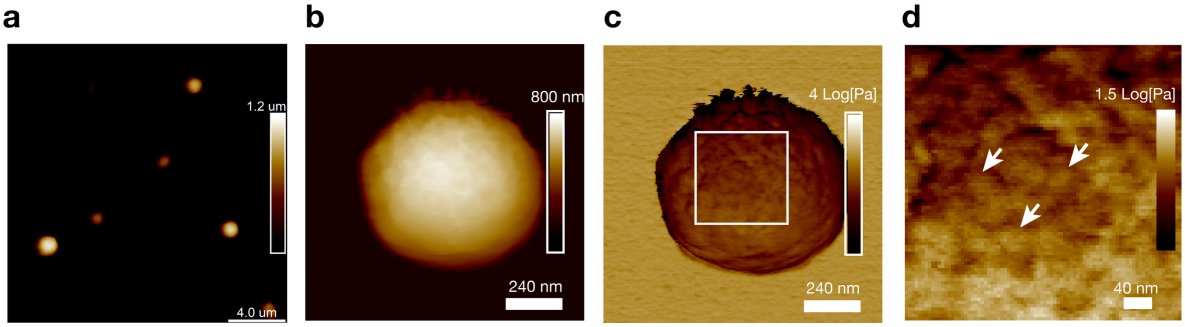

Supplementary Fig. 11 | Structural characteristics of CHD1 protein condensates. a, Large scale overview of a high-resolution (HR) AFM image of CHD1 condensates immobilized on a mica surface in 20 mM HEPES buffer. **b**, A representative HR-AFM topography image of a CHD1 protein condensate. **c**, Corresponding Young's modulus map, showing the internal structure of the condensate shown in **b**. **d**, A magnified AFM image captured from the framed region in **c**. Substructural features exhibiting different nanomechanical properties can be seen in both topography and nanomechanical maps (indicated by arrows in **d**). The CHD1 condensates consist of domains with different mechanical characteristics. The presence of different local domains with distinct elasticity might indicate that the CHD1 condensate consists of substructures with different protein concentrations, or components having different molecular interactions with RNA involved in the condensate formation, leading to the differences in their nanomechanical characteristics. The distinct mechanical properties of these local domains might result in different and specific interactions with the surrounding biomolecules.

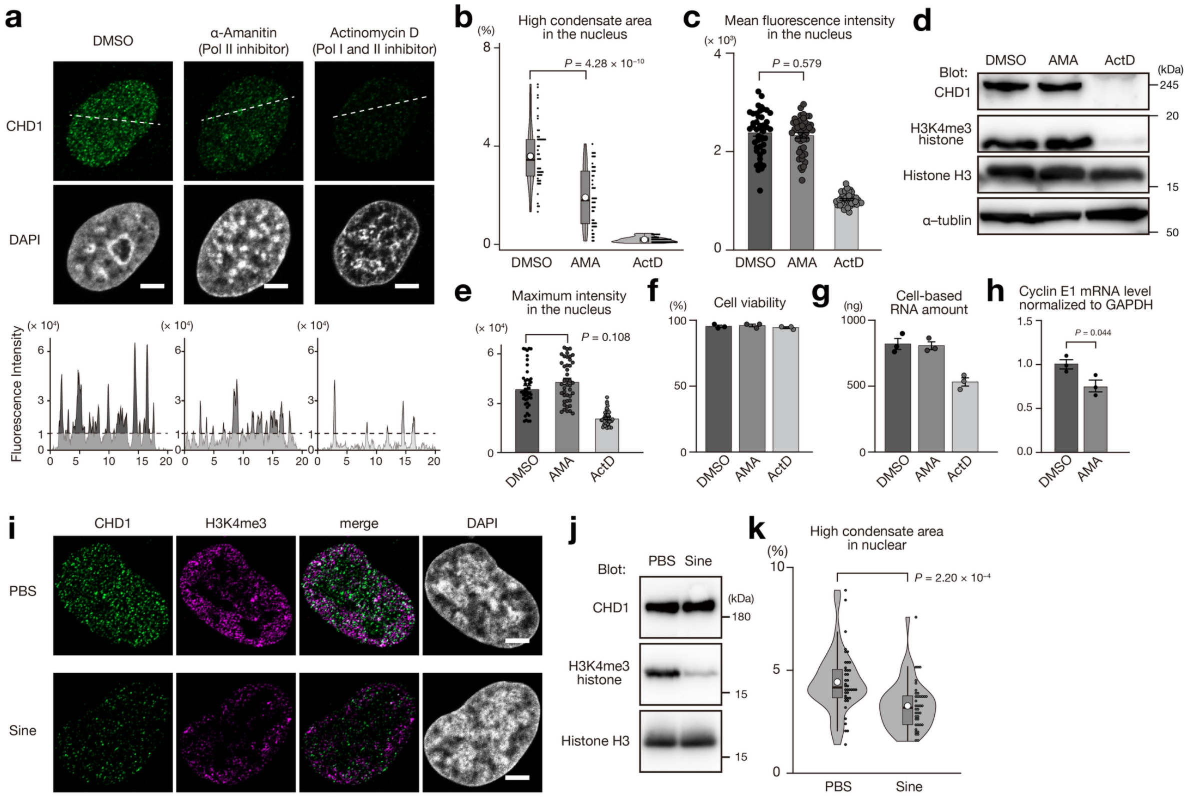

Supplementary Fig. 12 | Inhibition of RNA polymerases or histone methyltransferases leads to a reduction in CHD1 condensation within cells. a, Representative immunofluorescence images showing the CHD1 condensates under the effects of RNA polymerase II inhibition. Scale bar, 5 μm. The fluorescence intensity profiles (bottom) were taken along the white dashed line. High condensation areas of CHD1 were defined using a cutoff intensity of 1.0 × 10^4 (indicated by the dashed line). **b**, The percentage of high condensation areas of CHD1 in each nucleus was plotted as individual dots and analyzed using an unpaired Student's t-test. **c**, Mean fluorescence intensity of CHD1 for each nucleus was plotted as individual dots in data from more than 42 cells across three independent experiments. Unpaired student's t-test. **d**, Western blot analysis for CHD1 and H3K4me3. **e**, Maximum fluorescence intensity of CHD1 for each nucleus was plotted as individual dots. **f**,**g**,**h**, The effects of RNA polymerase II inhibition on cell viability (**f**), total cellular RNA amount (**g**), and relative Cyclin E1 mRNA expression (**h**). **i**, Representative immunofluorescence images showing the CHD1 condensates under the effects of sinefungin (Sine), a methyl-transferase inhibitor. Scale bar, 5 μm. **j**, Western blot analysis for CHD1 and H3K4me3 in the presence of sinefungin. **k**, Percentage of high condensation areas of CHD1 (a cutoff intensity of 6.0 × 10^4) in each nucleus was plotted as individual dots and analyzed using an unpaired Student's t-test (right). Source data are provided as a Source Data file.

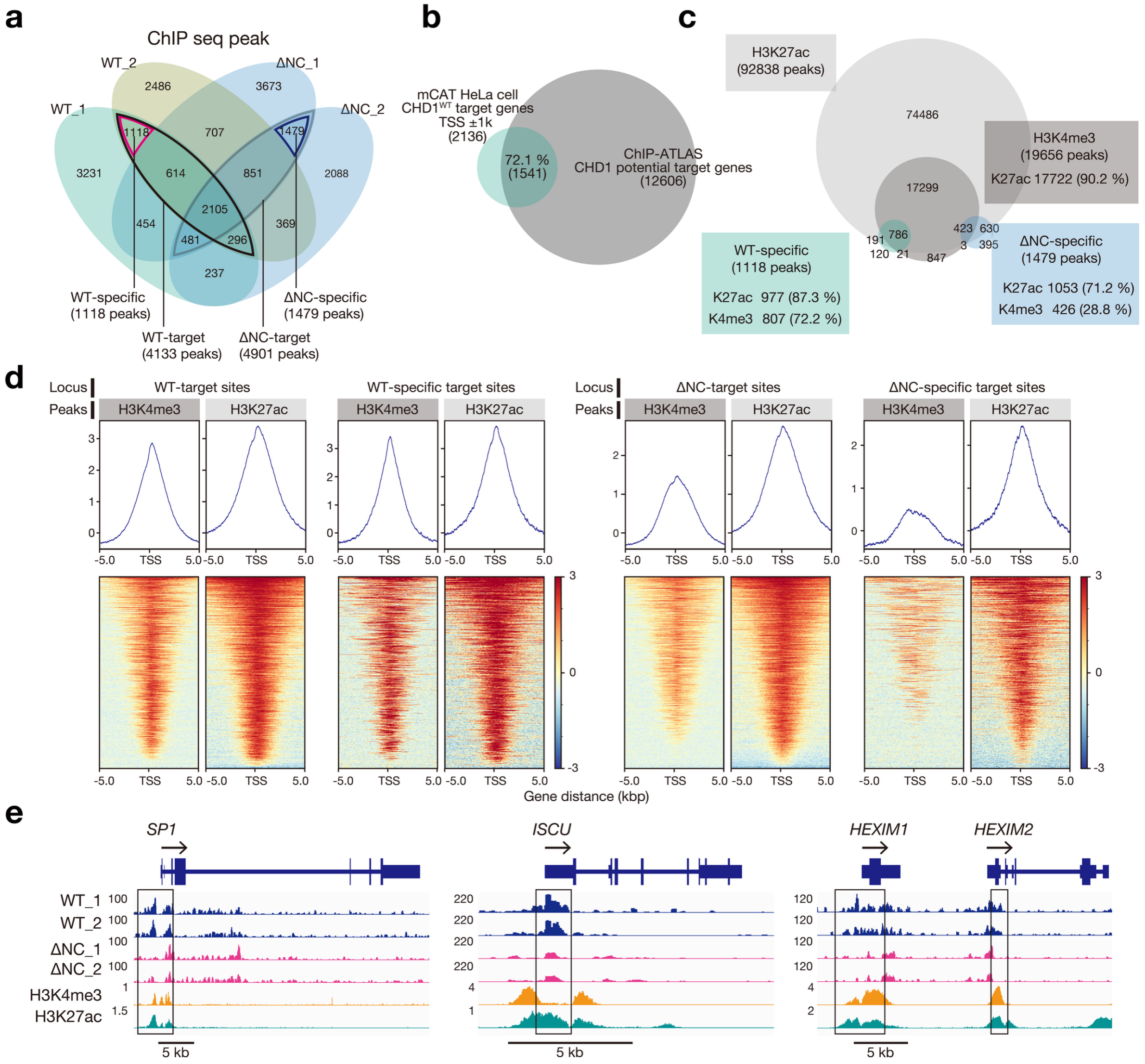

Supplementary Fig. 13 | ChIP-seq analysis of CHD1^{WT} and CHD1$^{\Delta NC}$. a, Venn diagram showing the overlap of ChIP-seq peaks in CHD1KO mCAT-HeLa cells reconstituted with CHD1^{WT} or CHD1$^{\Delta NC}$. The numbers indicate the total peaks. WT target: overlapping peaks between WT samples (*n* = 2). ΔNC target: overlapping peaks between ΔNC samples (*n* = 2). WT-specific: WT target peaks without ΔNC peaks. ΔNC-specific: ΔNC target peaks without WT peaks. **b**, Venn diagram illustrating the overlap between WT target genes and database-defined CHD1 potential target genes. WT target genes are defined as genes where CHD1 target peaks are located within ±1kbp of the transcription start site (TSS). **c**, Venn diagram presenting the overlap between WT-specific, ΔNC-specific, H3K27ac, and H3K4me3 peaks. The overlap between WT-specific peaks and H3K4me3 peaks was 72.2%, whereas the ΔNC-specific peaks exhibited a lower overlap at 28.8%. **d**, Heatmap

displaying ChIP-seq read densities of H3K4me3 and H3K27ac around TSS at WT target, WT-specific, ΔNC target, and ΔNC-specific sites. H3K4me3 peaks exhibited a higher enrichment in $CHD1^{WT}$ target genes when compared to $CHD1^{\Delta NC}$ target genes. **e**, ChIP-seq binding profiles of WT, ΔNC, H3K4me3, and H3K27ac on TP53-related genes [note the reduced ΔNC ChIP-seq signals at the TSS (boxed) of these gene loci]. Source data are provided as a Source Data file.

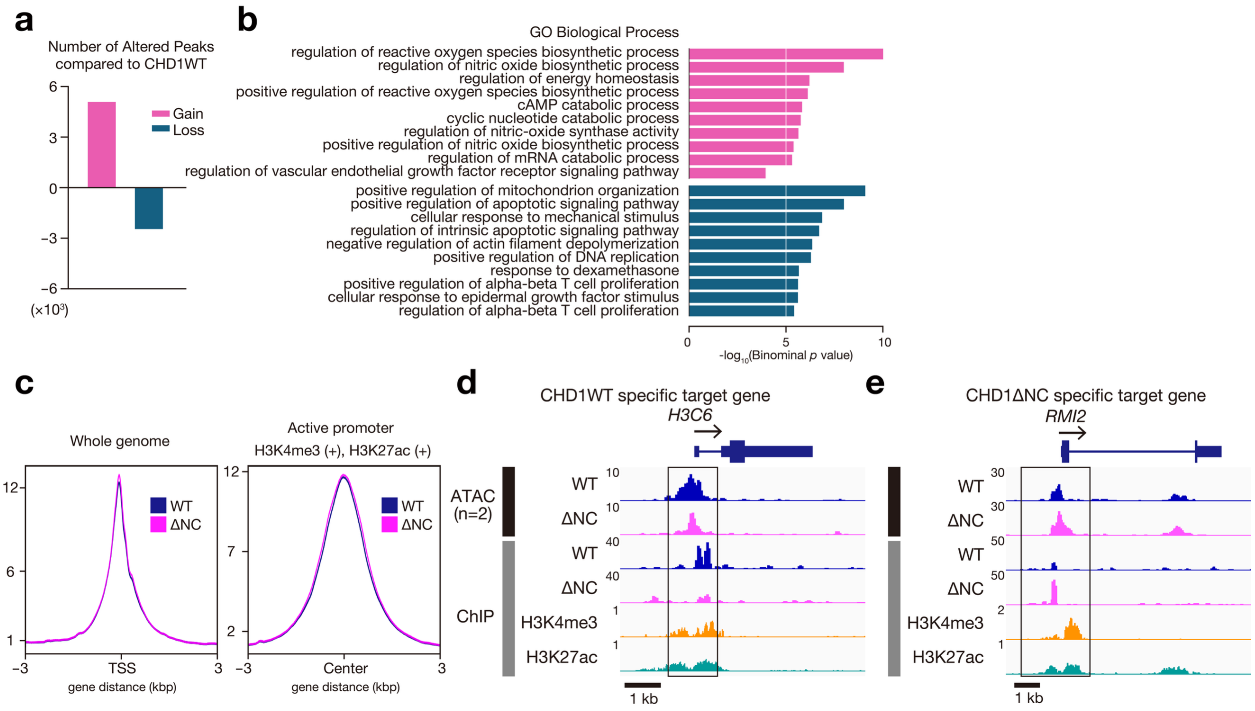

Supplementary Fig. 14 | Chromatin accessibility differences between CHD1^{WT} and CHD1$^{\Delta NC}$-expressing cells. a, Comparison of ATAC-seq peaks in CHD1$^{\Delta NC}$-expressing HeLa cells versus CHD1^{WT}-expressing HeLa cells. CHD1$^{\Delta NC}$-expressing cells exhibited a gain of 5,083 peaks and a loss of 2,455 peaks relative to CHD1^{WT}-expressing cells. **b**, Gene ontology analysis of the peaks gained or lost in CHD1$^{\Delta NC}$-expressing cells. **c**, Metaplot showing the distribution of ATAC-seq signal within a 3 kb window around TSS (left) and within active promoters (right) across the genome. **d**, **e**, Representative ATAC-seq peaks at target genes of CHD1^{WT} (**d**) and CHD1$^{\Delta NC}$ (**e**). Source data are provided as a Source Data file.

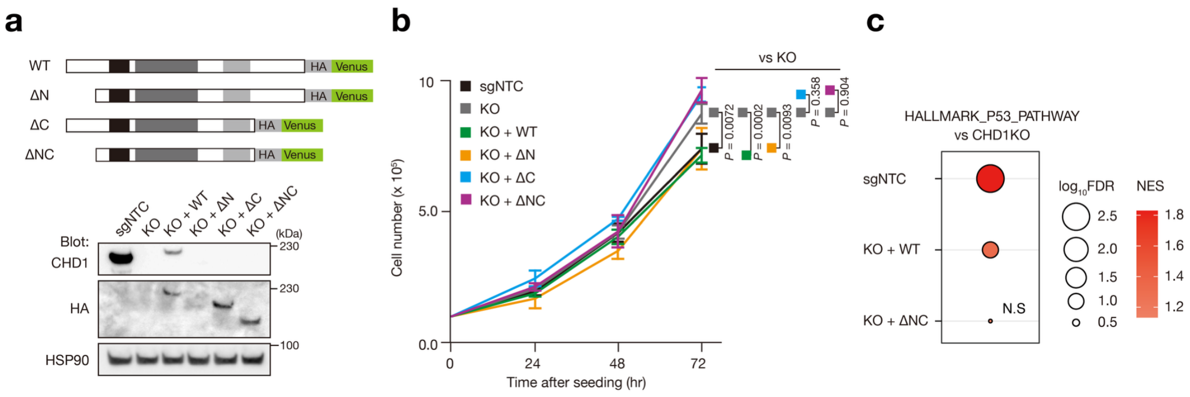

Supplementary Fig. 15 | IDR is essential for growth suppression. **a**, Schematic representation of HA-Venus-tagged CHD1^{WT} and IDR-deletion variants (upper panel). Western blot analysis confirmed the successful reconstitution of CHD1^{WT} and IDR-deletion mutants in CHD1 knockout (KO) mCAT-HeLa cells through retrovirus-mediated transduction (lower panel). An anti-CHD1 antibody recognizes the C-terminus deleted in ΔC and ΔNC. **b**, Growth curves of sgRNA nontargeting control (sgNTC) parental mCAT-HeLa cells, CHD1KO mCAT-HeLa cells, and KO cells reconstituted with either WT or IDR-deletion mutants. Data are presented as mean ± SEM (n = 3 independent experiments) and analyzed using two-way ANOVA. **c,** Balloon plots displaying GSEA results comparing the indicated groups with CHD1KO, highlighting the upregulation of the downregulated p53 pathway in CHD1KO cells upon restoration of CHD1^{WT}, but not by CHD1$^{\Delta NC}$. Balloon size and color indicate $\log_{10}$FDR and normalized enrichment score (NES), respectively. Source data are provided as a Source Data file.

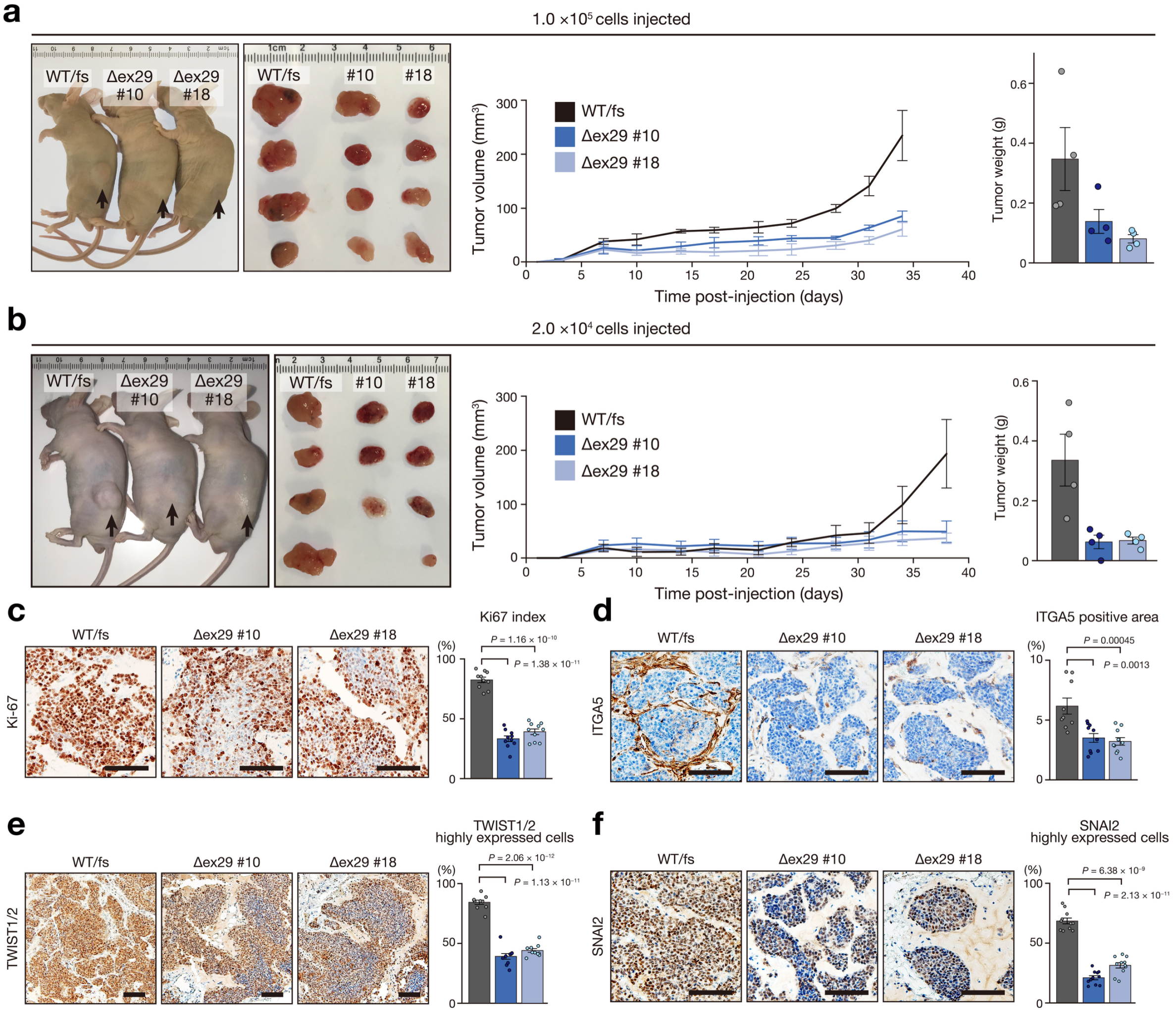

Supplementary Fig. 16 | 22RV1$^{\Delta ex29/\Delta ex29}$ clones exhibit reduced tumor growth and less pronounced EMT phenotypes in mice. a, **b**, Subcutaneous tumor growth of WT/fs 22Rv1 cells with sgRNA nontargeting control and Δex29/Δex29 clones in athymic nude mice (n = 4). 1.0 × 10^5 (**a**) and 2.0 × 10^4 (**b**) cells were injected. The right panels present tumor volume and weight as the mean ± SEM from four independent experiments. Differences in tumor volume and weight were analyzed using two-way ANOVA and unpaired Student's t-test, respectively. **c**−**f**, Representative immunohistochemistry images of tumor tissue for Ki-67 (brown, **c**) as a marker of proliferative cells, and integrin alpha 5 (ITGA5, brown, **d**) TWIST1/2 (brown, **e**) or SNAI2 (brown, **f**) as a marker of EMT. The tissues were counter stained with Mayer' s Hematoxylin and Eosin. Scale bars, 100 µm. The right panels present quantitative analysis of percentages of positive cells (Ki-67, TWIST1/2, or SNAI2) or positive area (ITGA5). Student's t-test. Source data are provided as a Source Data file.

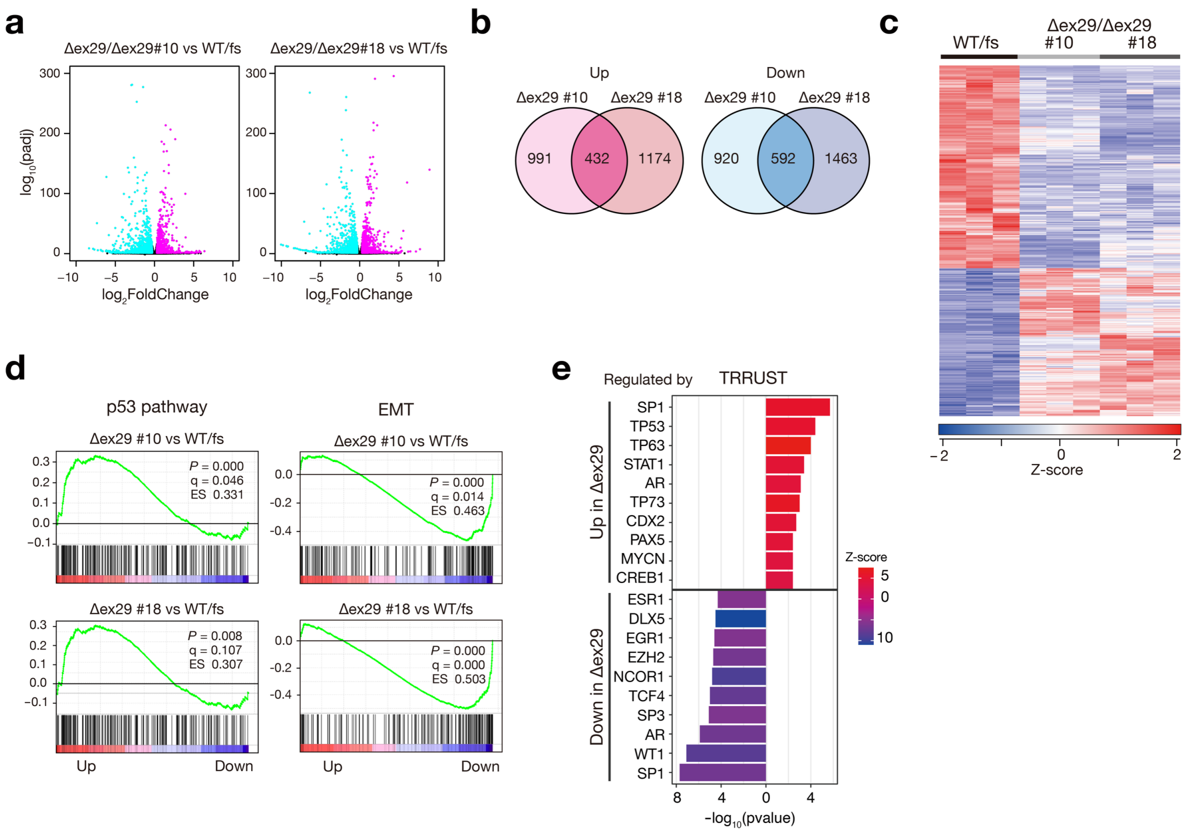

Supplementary Fig. 17 | The C-terminus-truncated CHD1 triggers oncogenic transcriptome. a, Volcano plots depicting gene expression profiles of Δex29/Δex29 clones compared to WT/fs 22Rv1 cells (*n* = 3 per genotype and clone). DEGs were determined using RNA-seq from three independent differentiation assays, with criteria of adjusted p-value (padj) < 0.05 and $\log_2$(fold change) > |0.3|. **b**, Venn diagrams showing the up-regulated or down-regulated genes in Δex29/Δex29 clones compared to WT/fs cells. **c**, Heat map displaying DEGs identified in WT/fs cells and Δex29/Δex29 clones (*n* = 3 per genotype and clone). **d**, GSEA demonstrated significant upregulation of the p53 pathway (left panel) and downregulation of the EMT (right panel) in Δex29/Δex29 clones compared to WT/fs cells. **e**, Enrichment analysis was performed using Metascape on DEGs (*n* = 3/group) to identify the top 10 enriched transcriptional regulatory relationships (TRRUST) associated with up-regulated or down-regulated genes in Δex29/Δex29 clones compared to WT/fs cells. Source data are provided as a Source Data file.

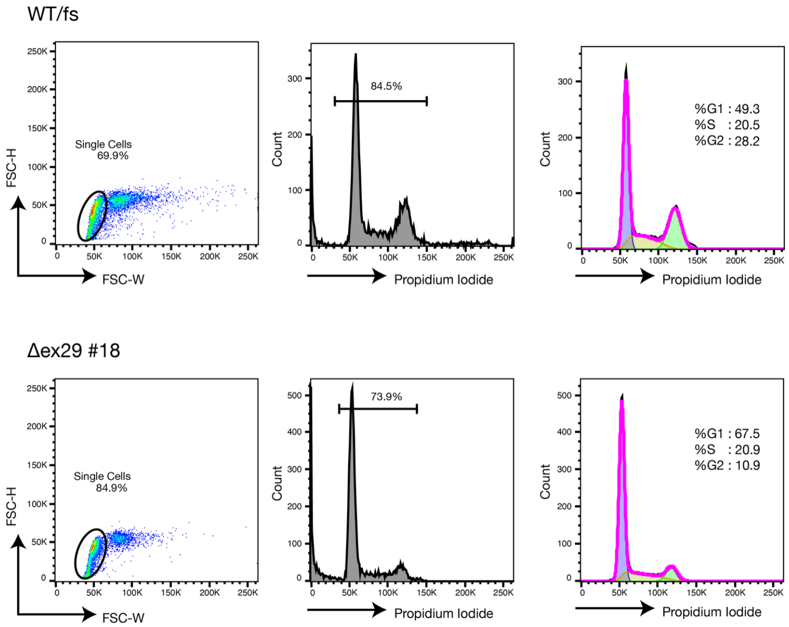

Supplementary Fig. 18 | Flow cytometry gating strategy for cell cycle analysis. Top: 22Rv1$^{WT/fs}$; bottom: 22Rv1$^{\Delta ex29/\Delta ex29}$

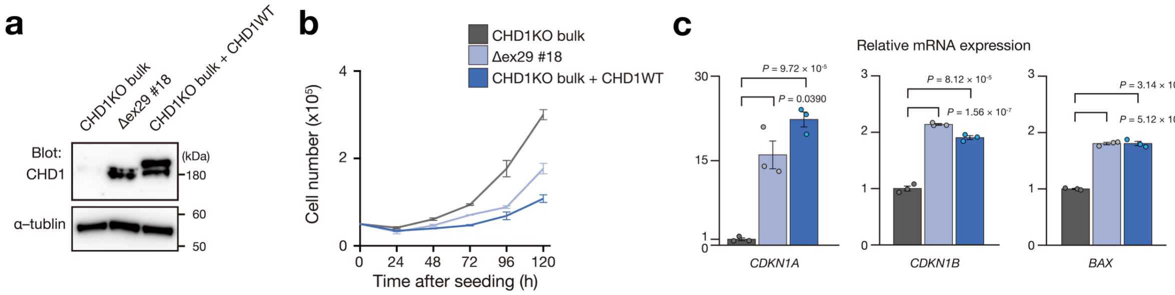

Supplementary Fig. 19 | Effect of CHD1$^{\Delta ex29}$ and CHD1^{WT} on cell growth and gene expression related to cell cycle inhibition. a, Western blot analysis showing CHD1 expression levels in 22Rv1$^{\Delta ex29/\Delta ex29}$ cells and CHD1KO 22Rv1 cells, with the latter transduced with either a puromycin resistance gene (CHD1KO bulk) or CHD1^{WT}-p2a-puro. **b**, Growth curves of 22Rv1 cells. **c**, RT-qPCR analysis of expression levels for *CDKN1A*, *CDKN1B* and *BAX*, normalized to *GAPDH*. Source data are provided as a Source Data file.

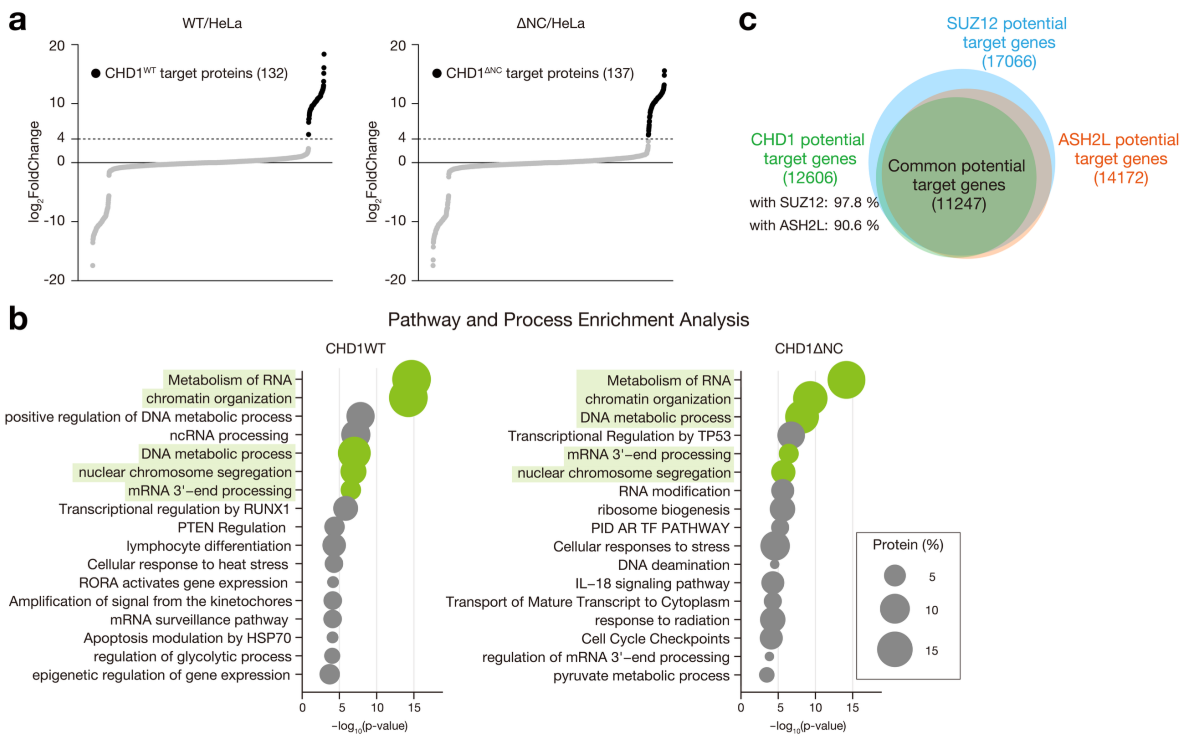

Supplementary Fig. 20 | Proximity-based labeling reveals CHD1 interacting proteins. a, Scatter plot of biotinylation M/S, comparing LFQ intensity of anti-GFP antibody in CHD1^{WT}–Venus or CHD1$^{\Delta NC}$–Venus restored HeLa cells compared to LFQ intensity of anti-GFP antibody in HeLa cells. CHD1 proximal proteins identified by $\log_2$WT/HeLa or ΔNC/HeLa > 4 are plotted in black. **b**, Pathway and process enrichment analyses of CHD1 proximal proteins using Metascape showing that several CHD1^{WT}- or CHD1$^{\Delta NC}$-interacting proteins participate in RNA metabolism, chromatin organization, DNA metabolic processes, nuclear chromosome segregation, and mRNA 3'-end processing. Common terms are highlighted in green. **c**, Venn diagram showing ChIP-Atlas defined potential target genes shared among CHD1, SUZ12, and ASH2L, indicating their colocalization at nearly identical genes. Source data are provided as a Source Data file.

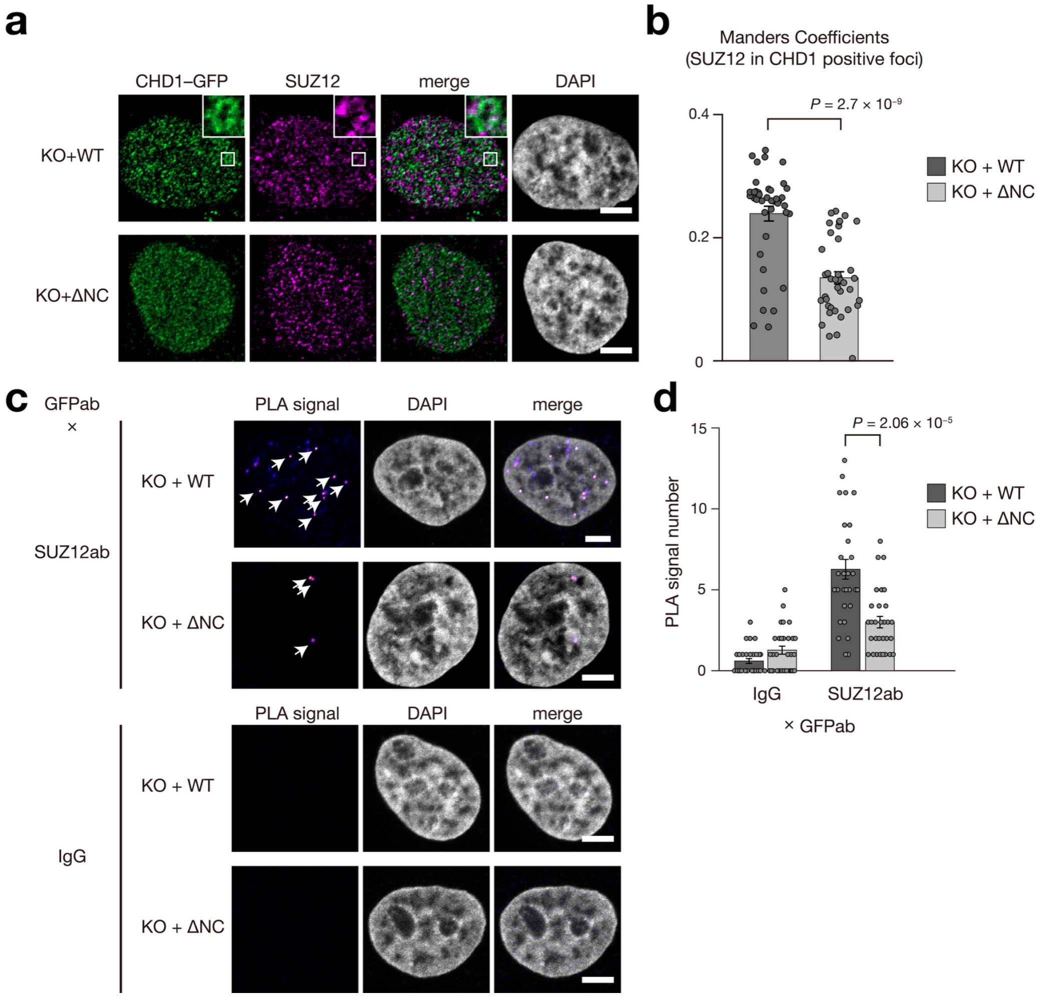

Supplementary Fig. 21 | Colocalization of CHD1 and SUZ12 condensates. a, Confocal immunofluorescence images showing the colocalization of CHD1 with SUZ12 in CHD1KO mCAT HeLa transduced to express $CHD1^{WT}$–Venus or $CHD1^{\Delta NC}$–Venus. Scale bars, 5 μm. **b**, Quantification of the CHD1 colocalization with SUZ12 in mCAT HeLa cells. Colocalized fractions of CHD1 were quantified based on Manders' coefficient. Data are means ± SEM of forty cells in at least two independent experiments. **c**, PLA using anti-GFP antibody and anti-SUZ12 antibody (top) or normal IgG (bottom) on CHD1KO mCAT HeLa cells expressing either $CHD1^{WT}$ or $CHD1^{\Delta NC}$–Venus. Scale bars, 5 μm. **d**, Quantification of PLA signals. Source data are provided as a Source Data file.

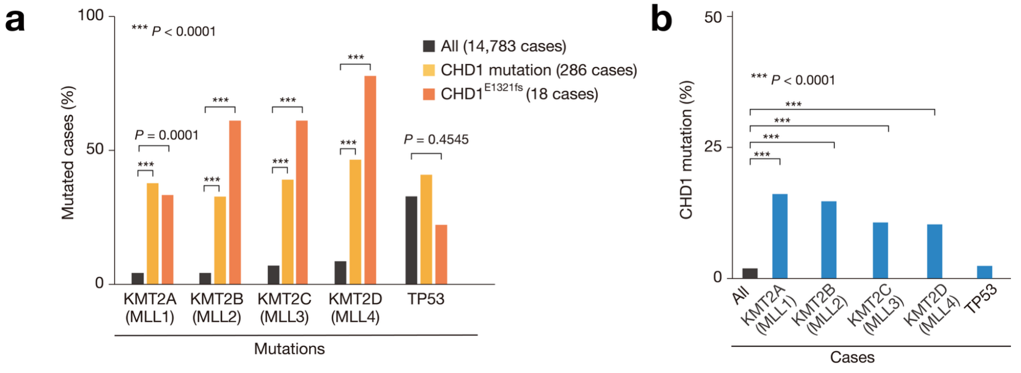

Supplementary Fig. 22 | Co-occurrence of mutations between CHD1 and KMTs in human cancers. a, Percentages of MLL/KMT2 family mutations and TP53 mutations in all cases across the NCI Genomic Data Commons, CHD1 mutations cases, and CHD1^{E1321fs} cases. TP53 is used as a standard. Two-tailed binominal test. **b**, Percentages of CHD1 mutations in all cases, MLL/KMT2 family and TP53 mutations cases. Two-tailed binominal test. Source data are provided as a Source Data file.

Uncropped scans of blots

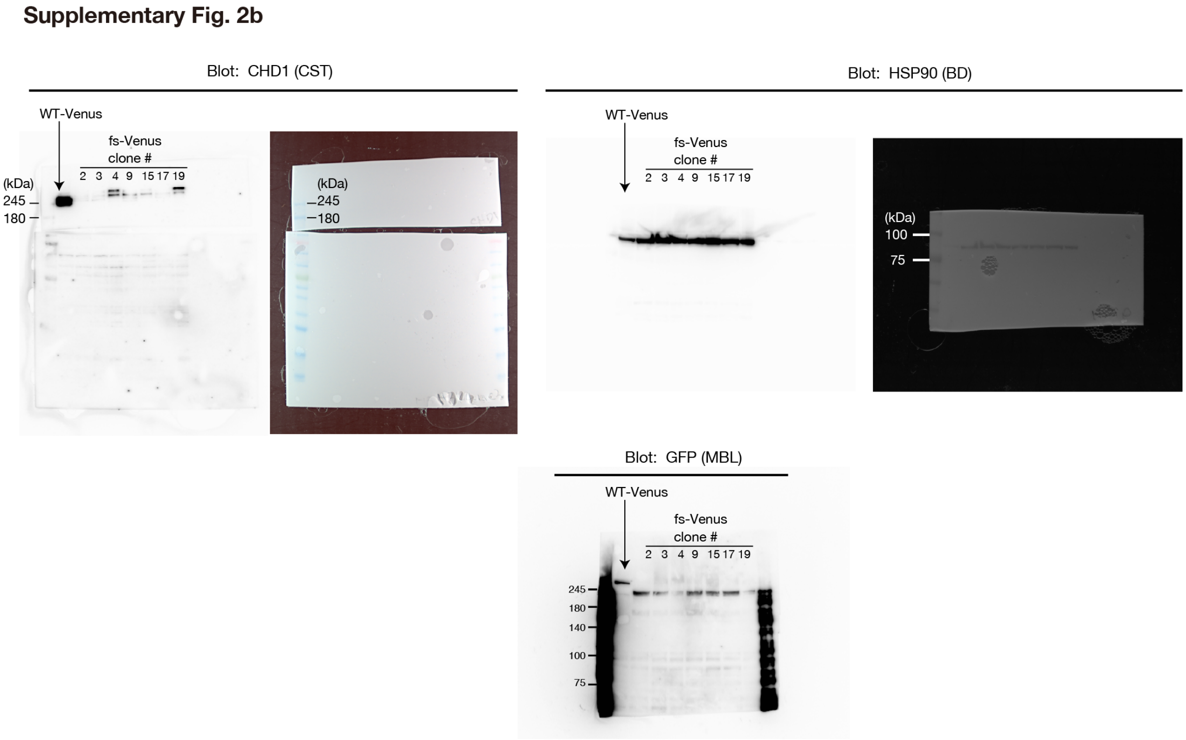

Supplementary Fig. 2b

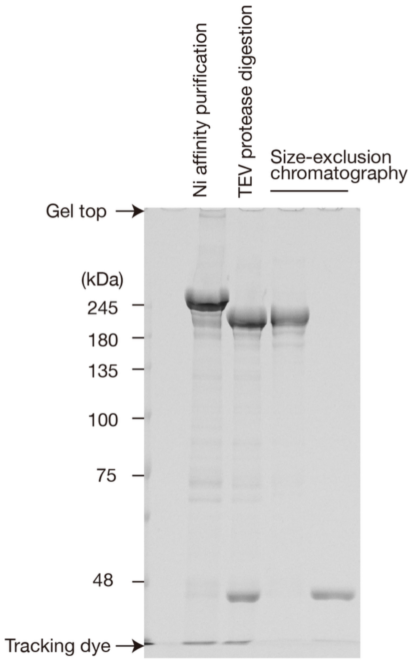

Supplementary Fig. 3b

Supplementary Fig. 5

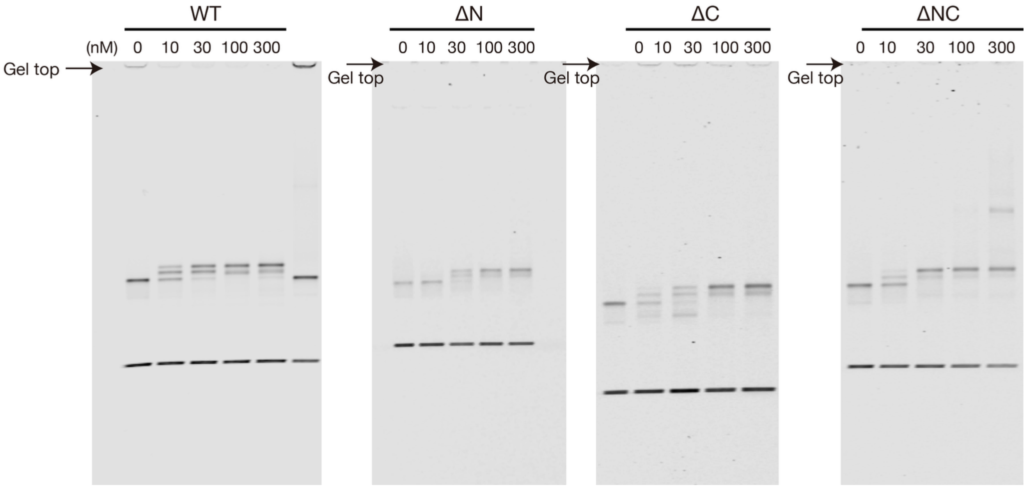

Supplementary Fig. 5

Supplementary Fig. 12d

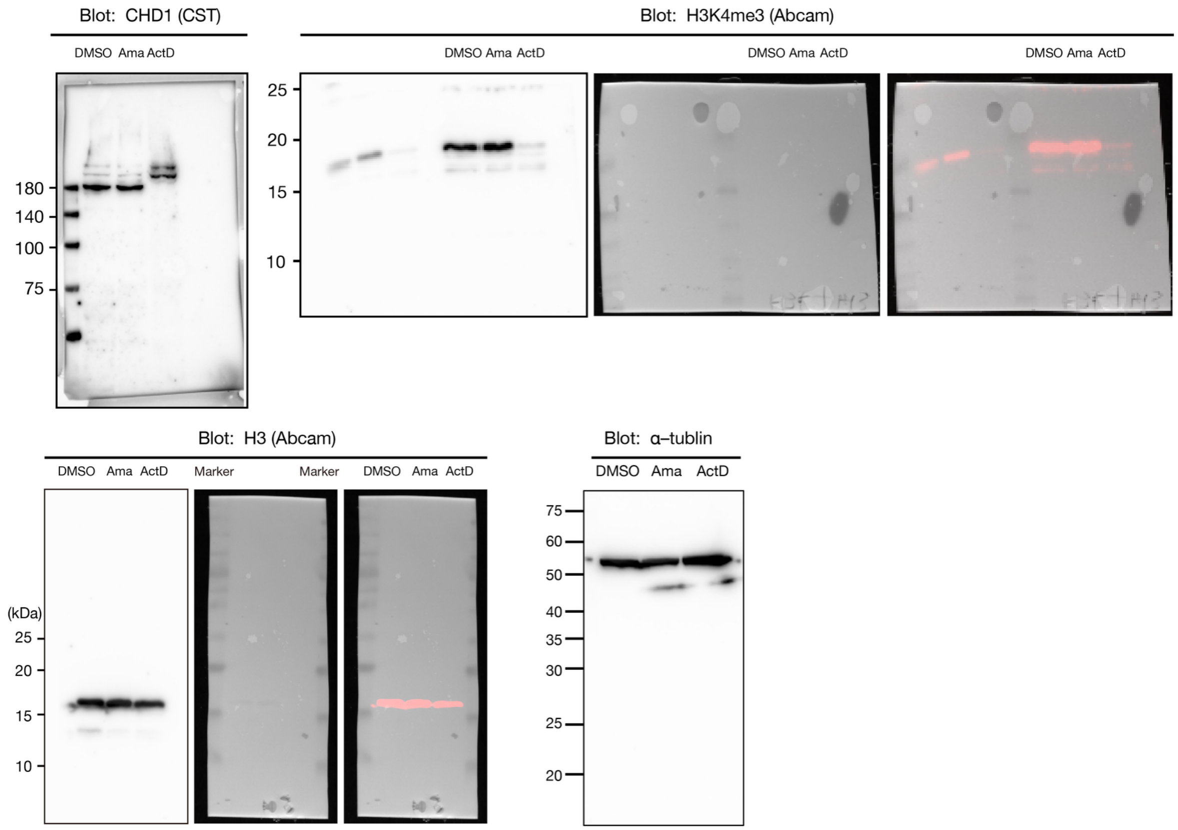

Supplementary Fig. 12g

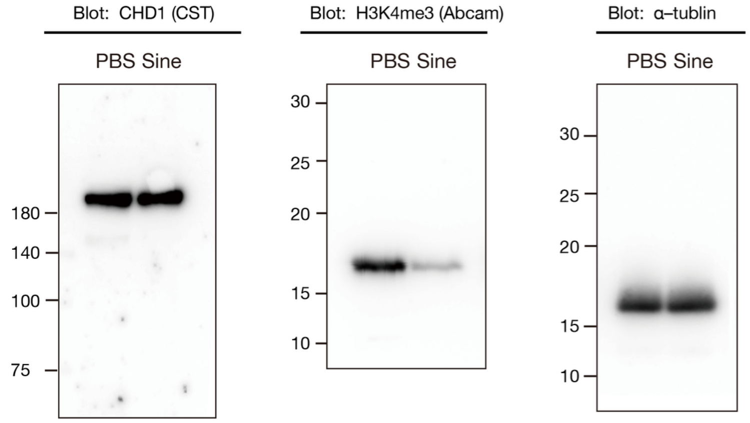

Supplementary Fig. 12d, g

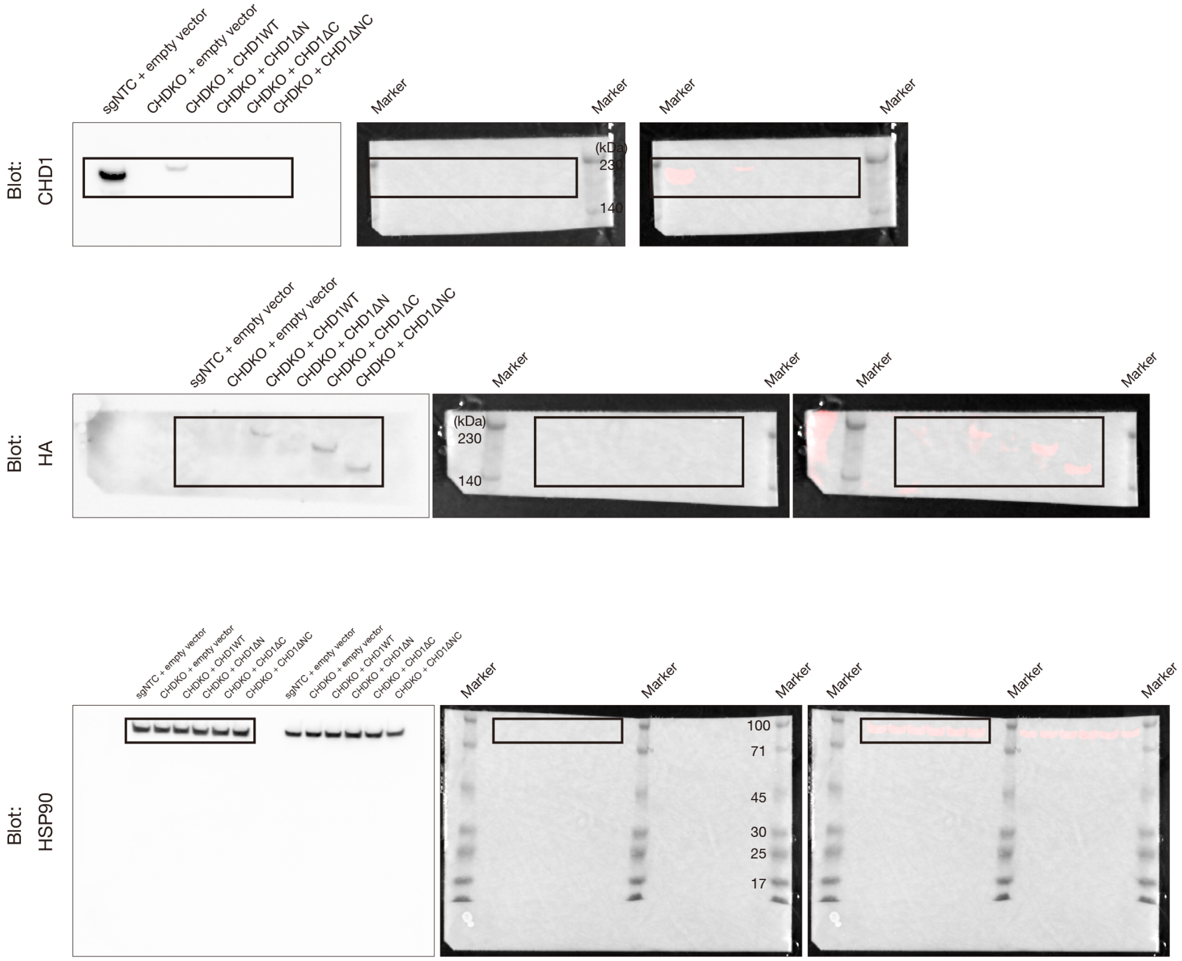

Supplementary Fig. 15a

Supplementary Fig. 18a

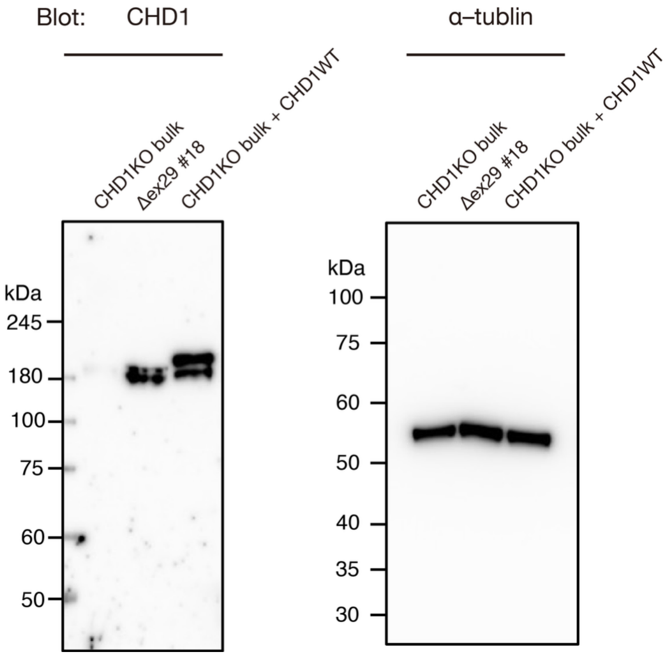

Supplementary Fig. 18a
