# Supplementary material for: Condensation-dependent interactome of a chromatin remodeler underlies tumor suppressor activities
Source: Nat Commun. 2025 Oct 30;16:9599. doi: 10.1038/s41467-025-64655-w (PMC12575849; doi:10.1038/s41467-025-64655-w)
Supplement: Supplementary file 2 — Description of Additional Supplementary Files [file 41467_2025_64655_MOESM2_ESM.pdf]

Description of Additional Supplementary Files

File Name: Supplementary Movie 1
Description: **HS-AFM single-molecule live imaging of CHD1WT**
The movie reveals the chromo domain, helicase/ATPase domain, and DNA-binding domain with globular morphologies, as well as intrinsically disordered regions at both the N- and C-termini and between the helicase/ATPase and DNA-binding domains. See Fig. 1e for a schematic representation. Scale bar, 10 nm.

File Name: Supplementary Movie 2
Description: **Live imaging of CHD1–Venus condensates undergoing deformation upon exposure to 1M NaCl in the droplet assay.**
Scale bar, 10 nm. Related to Supplementary Fig. 4e.

File Name: Supplementary Movie 3
Description: **Live imaging of CHD1–Venus condensates showing transformation and fusion events in the droplet assay.**
Scale bar, 10 nm. Related to Supplementary Fig. 4f.

File Name: Supplementary Movie 4
Description: **Confocal live imaging of Spot-FRAP analysis on CHD1WT–Venus KI HeLa cells.**
Scale bar, 5 µm. Related to Fig. 2e.
