# Supplementary material for: Condensation-dependent interactome of a chromatin remodeler underlies tumor suppressor activities
Source: Nat Commun. 2025 Oct 30;16:9599. doi: 10.1038/s41467-025-64655-w (PMC12575849; doi:10.1038/s41467-025-64655-w)
Supplement: Supplementary file 7 — Reporting Summary [file 41467_2025_64655_MOESM7_ESM.pdf]

nature portfolio

Corresponding author(s): Yasuhiro Tsukamoto
Katsuya Sakai
Yoshihiro Ogawa

Last updated by author(s): Sep. 20th, 2025

Reporting Summary

Nature Portfolio wishes to improve the reproducibility of the work that we publish. This form provides structure for consistency and transparency in reporting. For further information on Nature Portfolio policies, see our Editorial Policies and the Editorial Policy Checklist.

Please do not complete any field with "not applicable" or n/a. Refer to the help text for what text to use if an item is not relevant to your study.
For final submission: please carefully check your responses for accuracy; you will not be able to make changes later.

Statistics

For all statistical analyses, confirm that the following items are present in the figure legend, table legend, main text, or Methods section.

n/a	Confirmed	
☐	☒	The exact sample size (*n*) for each experimental group/condition, given as a discrete number and unit of measurement
☐	☒	A statement on whether measurements were taken from distinct samples or whether the same sample was measured repeatedly
☐	☒	The statistical test(s) used AND whether they are one- or two-sided<br>*Only common tests should be described solely by name; describe more complex techniques in the Methods section.*
☒	☐	A description of all covariates tested
☒	☐	A description of any assumptions or corrections, such as tests of normality and adjustment for multiple comparisons
☐	☒	A full description of the statistical parameters including central tendency (e.g. means) or other basic estimates (e.g. regression coefficient) AND variation (e.g. standard deviation) or associated estimates of uncertainty (e.g. confidence intervals)
☐	☒	For null hypothesis testing, the test statistic (e.g. *F*, *t*, *r*) with confidence intervals, effect sizes, degrees of freedom and *P* value noted<br>*Give P values as exact values whenever suitable.*
☒	☐	For Bayesian analysis, information on the choice of priors and Markov chain Monte Carlo settings
☒	☐	For hierarchical and complex designs, identification of the appropriate level for tests and full reporting of outcomes
☒	☐	Estimates of effect sizes (e.g. Cohen's *d*, Pearson's *r*), indicating how they were calculated

Our web collection on statistics for biologists contains articles on many of the points above.

Software and code

Policy information about availability of computer code

Data collection	Excluding the manufacturer's software required to run the instruments described in the Method section, no custom code was required to acquire any data in this manuscript.
Data analysis	Image processing: Fiji (version 2.14.0)<br>Statistics: GraphPad Prism 6.0<br>Prediction of intrinsically disordered regions: PONDR (http://www.pondr.com)<br>RNA-seq: FastQC (version 0.11.5), Trimmomatic (version 0.39), HISAT2 (version 2.2.1), Subread featureCounts (version 2.0.6), DESeq2 (version 1.38.3), GSEA software (version 4.3.2), Metascape (version 3.5), Enrichr (https://maayanlab.cloud/Enrichr/)<br>ChIP-seq: FastQC (version 0.11.5), Trimmomatic (version 0.39), Bowtie2 software (version 2.4.5), samtools (version 1.14), MACS peak caller (version 2.2.7.1), ChIPseeker (version 1.34.1), deeptools computeMatrix and plotHeatmap (version 3.5.1), bedtools (version 2.31.0)<br>ATAC-seq: FastQC (version 0.11.5), Trimmomatic (version 0.39), Bowtie2 software (version 2.4.5), samtools (version 1.14), MACS peak caller (version 2.2.7.1), ChIPseeker (version 1.34.1), deeptools computeMatrix and plotHeatmap (version 3.5.1), bedtools (version 2.31.0), GREAT (version 4.0.4)<br>High-speed AFM: Kodec4.4.7.39<br>High-resolution AFM: NanoScope Analysis software (version 1.9)<br>Biotinylation RNA-seq analysis: FastQC (version 0.11.5), TrimGalore (version 0.6.7), Bowtie2 software (version 2.4.5), samtools (version 1.14), Spiker split_bam.py script (version 1.0.3), featureCounts (version 2.0.6), LncSEA2.0 (https://bio.liclab.net/LncSEA/)<br>Mass-spectrometry: MaxQuant software (version 1.6.7.0), STRING (version 12.0)

For manuscripts utilizing custom algorithms or software that are central to the research but not yet described in published literature, software must be made available to editors and reviewers. We strongly encourage code deposition in a community repository (e.g. GitHub). See the Nature Portfolio guidelines for submitting code & softwar for further information.

Data

Policy information about availability of data

All manuscripts must include a data availability statement. This statement should provide the following information, where applicable:

- Accession codes, unique identifiers, or web links for publicly available datasets
- A description of any restrictions on data availability
- For clinical datasets or third party data, please ensure that the statement adheres to our policy

Data Availablity

The main data supporting the findings of this study are available within the Article and its Supplementary Information. The RNA-seq, ChIP-seq and ATAC-seq data are available at the Sequence Read Archive under accession numbers PRJNA1013253 (RNA-seq of 22Rv1 cells), PRJNA1013747 (RNA-seq of mCAT-HeLa cells), PRJNA1014223 (ChIP-seq of mCAT-HeLa cells), PRJNA1014249 (Biotinylation RNA-seq of mCAT-HeLa cells) and PRJNA1148813 (ATAC-seq of mCAT-HeLa cells).
Publicly available ChIP-seq datasets were reanalyzed in this study. Data were obtained from the DDBJ Sequence Read Archive under accession numbers DRR014675 and DRR014677 (H3K4me3 and H3K27ac ChIP-seq in HeLa cells, respectively).
Mass spectrometry raw data are available at the JPOST Repository under accession number (JPST004000).

Code Availablity

No custome code was used in this study.

Research involving human participants, their data, or biological material

Policy information about studies with human participants or human data. See also policy information about sex, gender (identity/presentation), and sexual orientation and race, ethnicity and racism.

Reporting on sex and gender	None
Reporting on race, ethnicity, or other socially relevant groupings	None
Population characteristics	None
Recruitment	None
Ethics oversight	None

Note that full information on the approval of the study protocol must also be provided in the manuscript.

Field-specific reporting

Please select the one below that is the best fit for your research. If you are not sure, read the appropriate sections before making your selection.

☒ Life sciences ☐ Behavioural & social sciences ☐ Ecological, evolutionary & environmental sciences

For a reference copy of the document with all sections, see nature.com/documents/nr-reporting-summary-flat.pdf

Life sciences study design

All studies must disclose on these points even when the disclosure is negative.

Sample size	No statistical method was used to predetermine sample sizes. Sample sizes were chosen to establish statistical significance on the basis of similar experiments reported in the literature or data from pilot experiments. Sample sizes were chosen as large as practically possible and adequate statistics have been applied.
Data exclusions	No data were excluded from the analyses.
Replication	Reproducibility was tested through multiple inter-experimental and intra-experimental replicates, as described per experiment in the paper.
Randomization	Mice were randomized into treatment groups. Each biochemical experiment in this study is rationally designed. Samples are not randomized for these experiments.
Blinding	Blinding was not used, as the design, execution and analysis of certain experiments were in many cases performed by a single investigator. This was necessary for data analysis and to minimize potential transposition errors.

Reporting for specific materials, systems and methods

We require information from authors about some types of materials, experimental systems and methods used in many studies. Here, indicate whether each material, system or method listed is relevant to your study. If you are not sure if a list item applies to your research, read the appropriate section before selecting a response.

Materials & experimental systems

n/a	Involved in the study
☐	☒ Antibodies
☐	☒ Eukaryotic cell lines
☒	☐ Palaeontology and archaeology
☐	☒ Animals and other organisms
☒	☐ Clinical data
☒	☐ Dual use research of concern
☒	☐ Plants

Methods

n/a	Involved in the study
☐	☒ ChIP-seq
☐	☒ Flow cytometry
☒	☐ MRI-based neuroimaging

Antibodies

Antibodies used

Rabbit polyclonal anti-CHD1 (Abcam, ab244391)
Rabbit monoclonal anti-CHD1 (Cell Signaling Technology, 4351)
Mouse monoclonal anti-CHD1 (Santa Cruz, sc-271626)
Rabbit polyclonal anti-GFP (MBL, 598)
Rabbit monoclonal anti-ASH2L (Cell Signaling Technology, 5019)
Rabbit monoclonal anti-SUZ12 (Cell Signaling Technology, 3737)
Mouse monoclonal anti-SUZ12 (Santa Cruz, sc-271325)
Rabbit polyclonal anti-H3K4me3 (Abcam, ab8580)
Mouse recombinant anti-RNA pol II CTD pSer5 (Active motif, 91119)
Mouse monoclonal anti-HSP90 (BD Biosciences, 610419)
Horse anti-mouse IgG (HRP conjugated) (Cell Signaling Technology, 7076)
Horse anti-rabbit IgG (HRP conjugated) (Cell Signaling Technology, 7074)
normal mouse IgG (Santa Cruz, sc-2025)
Goat polyclonal anti-mouse IgG HRP-conjugated antibody (R&D systems, HAF007)
Goat polyclonal anti-rabbit IgG HRP-conjugated antibody (R&D systems, HAF008,).
Alpha-tubulin (Sigma–Aldrich, T6074)
Rabbit monoclonal anti-Ki-67 (Abcam, ab16667)
Rabbit monoclonal anti-Integrin α5 (Abcam, ab150361) Mouse monoclonal anti-SLUG/SNAI2 (Santa Cruz, sc-166476) Rabbit polyclonal anti-TWIST1/2 (GeneTex, GTX127310)
Rabbit monoclonal anti-Vinculin (Cell Signaling Technology, 13901)

Validation

Commercial antibodies were validated by the manufacturer (see below link).
CHD1 (abcam): https://www.abcam.co.jp/products/primary-antibodies/chd1-antibody-ab244391.html
CHD1 (Cell Signaling): https://www.cellsignal.jp/products/primary-antibodies/chd1-d8c2-rabbit-mab/4351
CHD1 (Santa Cruz): https://www.scbt.com/ja/p/chd1-antibody-c-8
GFP: https://ruo.mbl.co.jp/bio/dtl/A/?pcd=598
ASH2L: https://www.cellsignal.jp/products/primary-antibodies/ash2l-d93f6-xp-rabbit-mab/5019
SUZ12 (Cell Signaling): https://www.cellsignal.jp/products/primary-antibodies/suz12-d39f6-xp-rabbit-mab/3737
SUZ12 (Santa Cruz): https://www.scbt.com/ja/p/suz12-antibody-d-10?srsltid=AfmBOooZMJWfff018adZWyzm7wVFYL3z33SmRuMUkPBX6DerlIX6XOqY
H3K4me3: https://www.abcam.co.jp/products/primary-antibodies/histone-h3-tri-methyl-k4-antibody-chip-grade-ab8580.html
RNA pol II CTD pSer5: https://www.activemotif.com/catalog/details/91119/abflex-rna-pol-ii-ctd-phospho-ser5-antibody-rab
HSP90: https://www.bdbiosciences.com/ko-kr/products/reagents/microscopy-imaging-reagents/immunofluorescence-reagents/purified-mouse-anti-hsp90.610419
Anti-mouse IgG (HRP conjugated): https://www.cellsignal.com/products/secondary-antibodies/anti-mouse-igg-hrp-linked-antibody/7076
Anti-rabbit IgG (HRP conjugated): https://www.cellsignal.jp/products/secondary-antibodies/anti-rabbit-igg-hrp-linked-antibody/7074
normal mouse IgG: https://www.scbt.com/p/normal-mouse-igg?srsltid=AfmBOoqcTNJfKT7I4cBw1vKgyoNHemjPtJL2XKiW1X_zA4Fy9M0YlqI8
Alpha-tubulin: https://www.sigmaaldrich.com/JP/ja/product/sigma/t6074?srsltid=AfmBOorWhVYqnynf4IKD2sfSClt-kaE9OHWYjGWbW8ocdyfwapUiIEDE
Ki-67: https://www.abcam.co.jp/products/primary-antibodies/ki67-antibody-sp6-ab16667.html
Integrin α5: https://www.abcam.co.jp/products/primary-antibodies/integrin-alpha-5-antibody-epr7854-ab150361.html
SLUG/SNAI2: https://www.scbt.com/ja/p/slug-antibody-a-7?srsltid=AfmBOoqZvOwNtf0_7ZZHar3YgAh5DubmP1rVFxvBNh2FwpJNTeVFWGw2
TWIST1/2: https://www.genetex.com/Product/Detail/Twist1-2-antibody/GTX127310?srsltid=AfmBOopRt3Hx8c6AaB5gkTdk90trHz-jc0XdFekHArQahR-Hb_Dom3yt
Vinculin: https://www.cellsignal.com/products/primary-antibodies/vinculin-e1e9v-xp-rabbit-mab/13901?srsltid=AfmBOoruezjQRL44nKZCbLen_mRnVCYKslLLZLBWYnHaR2gz35TbWZBP

Eukaryotic cell lines

Policy information about cell lines and Sex and Gender in Research

Cell line source(s)

22Rv1 cell line (ATCC) was gifted from I. Tamai (Kanazawa University, Ishikawa, Japan).
GP5d cell line was from ECACC. HeLa cell line was from ATCC.
mCAT-HeLa cells, and Plat-E cells were gifts from K. Nakayama (Kyushu University, Fukuoka, Japan). mCAT-HeLa cells and Plat-E were established as described in Yamaji, T et al.
Transmembrane BAX inhibitor motif containing (TMBIM) family proteins perturbs a trans-Golgi network enzyme, Gb3 synthase, and reduces Gb3 biosynthesis. J Biol Chem 285, 35505-35518 (2010).

Authentication

Only commercially obtained cell lines were authenticated by the vendor. All other cell lines used in this study were not independently authenticated by the authors.

Mycoplasma contamination

Cells were examined for possible mycoplasma contamination using the MycoAlert Mycoplasma Detection Kit (Lonza, LT07-118) and were negative.

Commonly misidentified lines
(See ICLAC register)

No commonly misidentified cell lines were used.

Animals and other research organisms

Policy information about studies involving animals; ARRIVE guidelines recommended for reporting animal research, and Sex and Gender in Research

Laboratory animals	Athymic nude mice (BALB/cAJcl-nu/nu, CLEA) were purchased from CLEA Japan.
Wild animals	This study did not involve wild animals.
Reporting on sex	This study did not involve wild animals.
Field-collected samples	The study did not involve field-collected samples.
Ethics oversight	All animal procedures were approved by Kanazawa University's Institutional Animal Care and Use Committee (IACUC). Mice were housed in AAALAC-accredited facilities and maintained on a 12 h light:12 h dark-light cycle with room temperatures of 21–23°C with 40–60% humidity. Tumour diameter and other endpoints did not exceed the limits permitted by IACUC. All mice were maintained under specific pathogen–free conditions.

Note that full information on the approval of the study protocol must also be provided in the manuscript.

ChIP-seq

Data deposition

☒ Confirm that both raw and final processed data have been deposited in a public database such as GEO.

☒ Confirm that you have deposited or provided access to graph files (e.g. BED files) for the called peaks.

Data access links<br>*May remain private before publication.*	https://dataview.ncbi.nlm.nih.gov/object/PRJNA1014223?reviewer=oebn7j83ev7r43qn9ig1q8e0ou
Files in database submission	Fastq file of mCAT-HeLa, CHD1KO plus CHD1WT-Venus, GFP_IP_replicate_1, ChIP-seq<br>Fastq file of mCAT-HeLa, CHD1KO plus CHD1WT-Venus, GFP_IP_replicate_2, ChIP-seq<br>Fastq file of mCAT-HeLa, CHD1KO plus CHD1ΔNC-Venus, GFP_IP_replicate_1, ChIP-seq<br>Fastq file of mCAT-HeLa, CHD1KO plus CHD1ΔNC-Venus, GFP_IP_replicate_2, ChIP-seq<br>Fastq file of mCAT-HeLa, CHD1KO plus CHD1WT-Venus, input, ChIP-seq<br>Fastq file of mCAT-HeLa, CHD1KO plus CHD1ΔNC-Venus, input, ChIP-seq
Genome browser session<br>(e.g. UCSC)	No longer applicable

Methodology

Replicates	Each sequencing of samples obtained through immunoprecipitation was with two replicates, and each sequencing of input was with one replicate.

Sequencing depth

Experiment	Total reads	mapped reads	paired or single
WT_IP_1	31,405,957	24,432,038	paired
WT_IP_2	32,024,125	27,131,903	paired
ΔNC_IP_1	32,108,728	28,455,343	paired
ΔNC_IP_2	34,659,460	28,411,550	paired
WT_input	36,288,349	33,822,838	paired
ΔNC_input	30,832,329	30,133,116	paired

Antibodies	Rabbit polyclonal anti-GFP (MBL, 598)
Peak calling parameters	macs2 callpeak -f BAMPE -p 1e-5 -B --gsize hs --nomodel --extsize 160
Data quality	FastQC (version 0.11.5) was run to check the sequencing quality.
Software	FastQC (version 0.11.5), Trimmomatic (version 0.39), Bowtie2 software (version 2.4.5), samtools (version 1.14), MACS peak caller (version 2.2.7.1), ChIPseeker (version 1.34.1), deeptools computeMatrix and plotHeatmap (version 3.5.1), bedtools (version 2.31.0)

Flow Cytometry

Plots

Confirm that:

☒ The axis labels state the marker and fluorochrome used (e.g. CD4-FITC).

☒ The axis scales are clearly visible. Include numbers along axes only for bottom left plot of group (a 'group' is an analysis of identical markers).

☒ All plots are contour plots with outliers or pseudocolor plots.

☒ A numerical value for number of cells or percentage (with statistics) is provided.

Methodology

Sample preparation	Cells were cultured at a density of 5 × 10^5 in a 6-well plate for 48 h. The 5 × 10^5 cells were harvested, and fixed with ice-cold 70% ethanol overnight at −20°C. Cells were centrifuged and washed twice with PBS, and then incubated in PBS containing propidium iodide (BD Pharmingen), 20 µg/ml Ribonuclease A (Nakalai 30141) and 0.1% Triton X-100 at 30°C in the dark for 30 min.
Instrument	BD FACS Canto II (BD Biosciences)
Software	Diva (BD Biosciences) software was used to collect data. Flowjo was used to analyze the flow data.
Cell population abundance	Cell sorting was not applied.
Gating strategy	FSC-A/SSC-A was used to gate cell population, and then single cells were gated based on FSC-H/FSC-A plot. Cells with abnormal DNA content (sub-G1 or >G2 DNA content), identified by Propidium Iodide staining, were excluded from downstream analyses.

☒ Tick this box to confirm that a figure exemplifying the gating strategy is provided in the Supplementary Information.

This checklist template is licensed under a Creative Commons Attribution 4.0 International License, which permits use, sharing, adaptation, distribution and reproduction in any medium or format, as long as you give appropriate credit to the original author(s) and the source, provide a link to the Creative Commons license, and indicate if changes were made. The images or other third party material in this article are included in the article's Creative Commons license, unless indicated otherwise in a credit line to the material. If material is not included in the article's Creative Commons license and your intended use is not permitted by statutory regulation or exceeds the permitted use, you will need to obtain permission directly from the copyright holder. To view a copy of this license, visit http://creativecommons.org/licenses/by/4.0/
